# Dioscin Ameliorates Hyperuricemia-Induced Atherosclerosis by Modulating of Cholesterol Metabolism through FXR-Signaling Pathway

**DOI:** 10.3390/nu14091983

**Published:** 2022-05-09

**Authors:** Ruixia Bao, Wei Wang, Beibei Chen, Jujie Pan, Qian Chen, Mengyang Liu, Dan Wang, Yuzheng Wu, Haiyang Yu, Lifeng Han, Yi Zhang, Tao Wang

**Affiliations:** 1 State Key Laboratory of Component-Based Chinese Medicine, Tianjin University of Traditional Chinese Medicine, 10 Poyanghu Road, Jinghai District, Tianjin 301617, China; bz93171125@163.com (R.B.); cbbzyzz@163.com (B.C.); liumengyang0212@tjutcm.edu.cn (M.L.); hyyu@tjutcm.edu.cn (H.Y.); hanlifeng@tjutcm.edu.cn (L.H.); zhwwxzh@tjutcm.edu.cn (Y.Z.); 2 Internal Medicine, University of Texas Medical Branch, 301 University Blvd, Galveston, TX 77555, USA; wewang2@utmb.edu; 3 Institute of Traditional Chinese Medicine, Tianjin University of Traditional Chinese Medicine, 10 Poyanghu Road, Jinghai District, Tianjin 301617, China; panjujie0309@163.com (J.P.); qianchen89@tjutcm.edu.cn (Q.C.); wyz2019@tjutcm.edu.cn (Y.W.); 4 Key Laboratory of Pharmacology of Traditional Chinese Medical Formulae (Tianjin University of Traditional Chinese Medicine), Ministry of Education, 312 Anshanxi Road, Nankai District, Tianjin 300193, China; wangdan0048@tjutcm.edu.cn

**Keywords:** dioscin, tigogenin, hyperuricemia, atherosclerosis, cholesterol, bile acid, FXR

## Abstract

Hyperuricemia is one of the independent risk factors for atherosclerotic cardiovascular disease. Herein, we investigate the association between uric acid and cholesterol metabolism and the effect of dioscin on the prevention of hyperuricemia-induced atherosclerosis. In the potassium oxonate-treated ApoE^−/−−/−^ mice, atherosclerosis was accelerated along with elevated serum cholesterol levels in the hyperuricemic state, which can be ameliorated by dioscin. Together with the in vitro assays, we found that the effect of dioscin was at least partially through the regulation of the farnesoid X receptor (FXR) -small heterodimer partner (SHP) -7α-hydroxylase (CYP7A1) signaling pathway in the liver. Tigogenin (a metabolite of dioscin) suppressed FXR activation and increased CYP7A1, resulting in an increased conversion rate of cholesterols into bile acids. Further clinical study revealed that treatment with a dioscin-enriched preparation decreased serum cholesterol levels in individuals with hyperuricemia. In summary, this study demonstrated a slowdown effect of dioscin on the progression of hyperuricemia-induced atherosclerosis.

## 1. Introduction

Hyperuricemia is defined as a serum uric acid level higher than 420 μmol/L (7.0 mg/dL) [1]. Epidemic studies have demonstrated that the prevalence of hyperuricemia in China was 13.3 %, among which, the male and female prevalence was around 19.4% and 7.9%, respectively [2]. The total affected population was up to 180 million in 2017. Hyperuricemia is one of the independent risk factors for arterial atherosclerosis, and has been known to increase the mortality of cardiovascular diseases [3,4]. The 2018 European guidelines for the treatment of high blood pressure highlighted that hyperuricemia is independently associated with the development of atherosclerotic cardiovascular disease [5].

Currently, the most common medications for hyperuricemia include xanthine oxidase inhibitor (XOI) (reducing the production of uric acid) and uricosurics that increase the excretion of uric acid. XOIs such as febuxostat decrease uric acid production by inhibiting xanthine oxidase [6]. However, compared with lifestyle modifications, 24 months of treatment with febuxostat did not delay the increase in intima-media thickness (IMT) of the common carotid arteries in asymptomatic hyperuricemia patients, demonstrated by a multicenter randomized controlled clinical trial with an endpoint of changes in the IMT of the carotid arteries [7].

Benzbromarone is a uricosuric medication, which increases the renal excretion of uric acid [8]. A cohort study on patients using allopurinol (*n* = 103,695) or benzbromarone (*n* = 20,739) showed that the allopurinol group had a 22% (PS-matched HR 1.22, 95% CI 1.05–1.41) higher risk for cardiovascular endpoints than the benzbromarone group during an average 1.2-year follow-up. However, the severe hepatotoxicity of benzbromarone limited its long-term use [9].

Hyperuricemia is commonly associated with dyslipidemia [10]. Studies on the pathological mechanisms of atherosclerosis have suggested that the malfunction of lipid metabolism, especially the imbalance of cholesterol metabolism, is one of the main risk factors for atherosclerosis [11,12]. Cholesterol metabolism in humans generally consists of four processes: intrinsic synthesis, extrinsic absorption, excretion, and esterification [13]. Malfunction of any of these processes can lead to elevated cholesterol levels. To date, the mechanism of uric acid-induced dyslipidemia has not been fully elucidated. Some clinical studies have demonstrated that statins, known to inhibit the intrinsic synthesis of cholesterols, lowered the cholesterol levels in hyperuricemia patients. However, this does not necessarily suggest a correction of uric acid-induced dysfunction of cholesterol metabolism because intrinsically synthesized cholesterols account for two-thirds of total cholesterols. Therefore, the TC level would decrease whenever the intrinsic synthesis was inhibited. On the other hand, the intrinsic synthesis of cholesterols is involved in multiple physiological functions such as hormone metabolism, nutrition absorption, and maintenance of cellular structures [14,15]. Hence, using cholesterol synthesis inhibitors for uric acid-induced dysfunction of cholesterol metabolism carries potential unpredictable risks as the mechanisms of association between the two disorders remain unclear. These clinical results suggested: (1) uric acid is not a direct risk factor for cardiovascular diseases; (2) uricosuric medications are likely superior to the XOIs in reducing cardiovascular risks if adverse effects are not considered.

Accordingly, we emphasize the importance of modulating uric acid excretion in managing uric acid-induced dysfunction of cholesterol metabolism. We suggest the use of pertinent medications based on the mechanisms of uric acid-induced dyslipidemia, which would more effectively reduce the risks of cardiovascular events in hyperuricemia patients.

Our previous studies showed that dioscin significantly lowered the uric acid level in hyperuricemic mice [16]. Tigogenin, the active metabolite of dioscin, reduced uric acid levels by increasing the renal excretion of uric acid through inhibiting the uric acid reabsorption protein urate reabsorption transporter 1, and enhancing intestinal excretion of uric acid through promoting intestinal transporter ATP-binding cassette transporter. Meanwhile, it has been reported by other groups that dioscin improved lipid metabolism and reduced atherosclerosis by modulating oxidative stress and inflammation [17].

In this study, we investigated the potential effects of dioscin in modulating uric acid-induced dysfunction of cholesterol metabolism, and the subsequently reduced risks of atherosclerosis associated with hyperuricemia. We demonstrated that dioscin decreased cholesterol levels through modulating the steady state of bile acids by inhibiting farnesoid X receptor (FXR), and increased the synthesis of bile acids.

## 2. Materials and Methods

### 2.1. Materials

Dioscin was obtained from MedChemExpress (Monmouth Junction, NJ, USA). The high cholesterol diet (HCD, containing 0.5% of cholesterols and 21% of fat) was obtained from MediScience Diets Co., Ltd. (Yangzhou, China). Tigogenin and various bile acids standards were purchased from Shanghai Yuanye Biotechnology Co., Ltd. (Shanghai, China, purity >95% by HPLC method). The internal standard, nordeoxycholic acid (NDCA) was obtained from Steraloids, Inc. (Newport, RI, USA). Potassium oxonate (PO), uric acid, and adenine were purchased from Sigma-Aldrich (St. Louis, MO, USA).

### 2.2. Animal

All animal experimental designs were approved by the Science and Technological Committee and the Animal Use and Care Committee of TJUTCM (No. TCM-LAEC2021010). Eight-wee-old male C57BL/6J mice were purchased from Beijing Vital River Laboratory Animal Technology Co., Ltd. (Beijing, China). ApoE deficient (ApoE^−/−^) mice, male, SPF grade, 8 weeks old, were purchased from Beijing HFK Bioscience Co., Ltd. (Beijing, China). All animals had free access to standard diet and water and were housed in experimental conditions at 25 ± 2 °C with humidity of 60 ± 5% in a fixed 12 h artificial light period.

The C57BL/6J mice were randomized into the normal control group (*n* = 8) and hyperuricemia group (*n* = 8). The mice of the hyperuricemia group received PO (200 mg/kg body weight) and adenine (50 mg/kg body weight) through gastric gavage, and the normal control mice received saline with the same volume. The treatment was applied once a day in the morning for 21 consecutive days. At the end of the administration, mice were euthanized by an overdose of isoflurane; blood and liver samples were collected and stored at −80 °C until analysis.

ApoE^−/−^ mice were randomly allocated into the control ApoE^−/−^ mice group (*n* = 10), PO-induced hyperuricemic ApoE^−/−^ mice group (*n* = 10) and dioscin-treated hyperuricemic ApoE^−/−^ mice group (*n* = 10). All ApoE^−/−^ mice were fed with an HCD. The hyperuricemic ApoE^−/−^ mice group (dose: 250 mg/kg, volume: 20 mL/kg, in saline) was treated with PO intraperitoneal injection once a day and the control ApoE^−/−^ mice received saline injection with the same volume continuously for 3 months. The dioscin-treated hyperuricemic ApoE^−/−^ mice received daily treatment of dioscin (100 mg/kg body weight) through gastric gavage for 3 months. At the end of the administration, mice were euthanized by an overdose of isoflurane blood; liver, ileum, and the main artery were quickly and carefully dissected on an ice plate. All specimens were stored at −80 °C for further analysis.

### 2.3. Cell Culture and Treatment

The hepatic cell line HepG2 (ACC, No. 85011430, ECACC, Salisbury, UK) was maintained in Minimum Essential Medium supplemented with 10% fetal bovine serum, 1% non-essential amino acids, and 1% penicillin–streptomycin under a humidified atmosphere of 5% CO_2_ in the air. Cells were seeded at 2 × 10^5^ cells/mL on a 6-well plate. After growth to 80% confluence, cells were treated with chenodeoxycholic acid (CDCA) (50 μM) alone or CDCA (50 μM) combined with tigogenin (20 μM). After 24 h incubation, cells were harvested for quantitative real-time polymerase chain reaction (qRT-PCR) analysis.

### 2.4. Uric Acid and Lipids Analysis

UPLC analysis was used to determine the serum uric acid level of mice as described previously [18]. The total cholesterol (TC), high-density lipoprotein cholesterol (HDL-C), and low-density lipoprotein cholesterol (LDL-C) levels of serum were determined with the commercial kits (BioSino Biotechnology and Science Inc., Beijing, China) according to the manufacturer’s instructions. Hepatic TC and triglycerides (TG) were extracted using the previous method [19] and then were determined with the same commercial kits described above.

### 2.5. Histological Oil Red O Staining

Liver sections were fixed in 10% formalin overnight, embedded in paraffin, sectioned, and then stained with Oil Red O, according to the manufacturer’s protocol. Images of the sections were obtained with Axio Imager D2 (Zeiss, Oberkochen, Germany).

### 2.6. Atherosclerotic Lesions Analysis

Aortic lesions were determined as previously described [20]. Briefly, after euthanasia, the mouse whole aorta was harvested. The aorta was then fixed in 4% paraformaldehyde. After removal of outside connecting tissue and fat, the aorta was stained with Oil Red O solution, and lesions in en face aorta were quantified by computer-assisted image analysis, and calculated as percent lesion area. To determine the sinus lesions in the aortic root, 5 μm frozen sections of the aortic root were collected and stained with Oil Red O solution. Images of the sections were obtained with Axio Imager D2 (Zeiss, Oberkochen, Germany). The total morphometric lesion area was determined with a computer-assisted image analysis procedure.

### 2.7. Bile acid Profile Analysis

Serum samples of hyperuricemia individuals or mice (100 μL) were mixed with 1 mL ice-cold acetonitrile, and then vortexed vigorously. After keeping on ice for 30 min, the mixture was centrifuged at 14,000× g for 10 min under 4 °C. The supernatant was evaporated under nitrogen flow. The residue was re-dissolved in 50 μL of 100% methanol, and centrifuged at 20,000× g for 10 min under 4 °C. The supernatant was used for liquid chromatography–mass spectrometry (LC-MS) analysis.

For liver and ileum samples of mice, approximately 50 mg liver and 20 mg ileum were homogenized in 5 volumes of ddH_2_O, NDCA (100 ng) was added as an internal standard to 250 μL of tissue homogenate, and vortexed thoroughly. The homogenate was then added with 1.25 mL of ice-cold acetonitrile, and shaken vigorously. Afterward, the processing steps were the same as serum samples.

Detection and quantification were achieved using the Waters UPLC system (Waters Corporation, Milford, MA, USA) coupled to an Exactive™ Plus Orbitrap mass spectrometer equipped with an electrospray ionization (ESI) source (ThermoFisher Scientific, Waltham, MA, USA). 

The separation was accomplished on a Waters ACQUITY UPLC BEH C18 column (1.7 μm, 2.1 × 100 mm, Waters Co, Milford, MA, USA). The column temperature was 55 °C, the flow rate was 0.4 mL/min and the injection volume was 2 μL. The mobile phase consisted of 0.01% acetic acid in acetonitrile: H_2_O (80:20, mobile phase A) and 0.01% acetic acid in acetonitrile: H_2_O (20:80, mobile phase B). The gradient elution of the mobile phase was shown in Supplementary Table S1.

The setting parameters of the mass spectrometer were: 4 L/min for try gas flow; ESI source was operated in the negative mode; 0.4 Bar for nebulizer; 3.5 KV for spray needle voltage; 190 °C for dry gas; 15 eV for collision energy; ion analysis was performed using the selected ion monitor mode; acquisitions were performed using the MS/MS mode, and the measurement range was MS m/z 100–1500; MS^2^ m/z 50–1500; and MS^3^ m/z 50–1500. The data acquisition and analysis were carried out using Xcalibur™ Software 4.0. Twelve kinds of BA were prepared in methanol, including cholic acid (CA), CDCA, deoxycholic acid (DCA), lithocholic acid (LCA), ursodeoxycholic acid (UDCA), taurochenodeoxycholic acid (TCDCA), tauroursodeoxycholic acid (TUDCA), taurocholic acid (TCA), α-muricholic acid (αMCA), β-muricholic acid (βMCA), tauro-α-muricholic acid (TαMCA), and tauro-β-muricholic acid (TβMCA) were used as standard samples.

### 2.8. QRT-PCR

RNA isolation, cDNA synthesis, and qRT-PCR analysis were performed as described previously [18]. The primers used for qRT-PCR were synthesized by Dingguo Bio Co. Ltd., Shanghai, China. Sequences used for qRT-PCR were shown in Supplementary Table S2 (primer for mice) and Supplementary Table S3 (primer for HepG2 cells). Results were presented as levels of expression relative to those of controls after normalization to GAPDH using the 2−^△△^^CT^ methods. Analysis was carried out in triplicates.

### 2.9. FXR Mediated Coactivator Recruitment Assay

FXR coactivator recruitment assay was performed according to the manufacturer’s instructions (Invitrogen, Waltham, MA, USA). The activity of FXR was determined in Black, 384-well assay plates from PerkinElmer Life Sciences. FXR-LBD (5 nM) was added to the test compounds followed by the addition of a mixture of the fluorescein-coactivator peptide (500 nM) and terbium anti-GST antibody (5 nM); CDCA was included in assays as the positive controls. After an incubation period at room temperature, the fluorescence emission signals were detected at 520 nm and 495 nm. The TR-FRET ratio of 520:495 was used for the calculation of the EC_50_ and IC_50_ using the curve-fitting software GraphPad Prism version 8.0 (San Diego, CA, USA).

### 2.10. Clinic Trial of Dioscin-Enriched Preparation in Hyperuricemia Individual

A randomized controlled trial was conducted at Metabolic Diseases Hospital, Tianjin Medical University, Tianjin, China, from September 2017 to December 2018, in accordance with the International Council for Harmonization guidelines. Each subject provided written informed consent before participation. The trial was approved by the Tianjin Medical University (DXBYYhMEC2017-23-2) and registered with the Chinese Clinical Trial Registry (number ChiCTR-IPR-17014035).

Sixty adults aged 18–60 with fasting serum uric acid levels higher than 420 μmol/L were enrolled. Participants were excluded if they had serious cardiovascular, respiratory, excretory, digestive, mental illness, or gout. After inclusion, the participants were randomized into two arms, which were the placebo group and the dioscin-enriched preparation (commercial name: Di’ao Xinxuekang Capsule) (DA) group. DA group received 600 mg of DA per day for one month, and the control group received a placebo. DA was a kind gift from Di’ao Group Chengdu Pharmaceutical Co. Ltd. (100 mg/capsule, lot: 1701020.) Serum uric acid and cholesterol levels were determined.

### 2.11. Statistical Analysis

SPSS 20.0 statistical software (version 20, SPSS; IBM, Armonk, NY, USA) was used to perform statistical analysis. Data were expressed as the mean ±S.E.M. Shapiro–Wilk test was used to assess the normal distribution of variables. Pearson’s correlation analyses were used to evaluate the correlation between the changes in serum uric acid and TC. Paired *t*-test was used to compare the differences between day 0 and one month later. For two independent (unrelated) groups, an independent *t*-test was used to compare the differences. For three or more independent (unrelated) groups, significant differences were evaluated by one-way analysis of variance (ANOVA); LSD and Dunnett’s tests were used for post hoc evaluations with the normal distribution of variables. Kruskal–Wallis test was used to assess differences with non-normally distributed data. *p* < 0.05 was considered to represent a statistically significant difference.

## 3. Results

### 3.1. Hepatic CYP7A1 Expression Was Reduced in Hyperuricemia Mice Associated with an Elevation in Serum Cholesterol Levels

As previously reported, hyperuricemia can induce dysfunction of cholesterol metabolism, which was considered a key factor in the occurrence and development of atherosclerosis. However, the mechanism is unclear. Firstly, PO-treated mice were used to clarify the pathophysiological linkage between uric acid and cholesterol.

**Figure 1:**
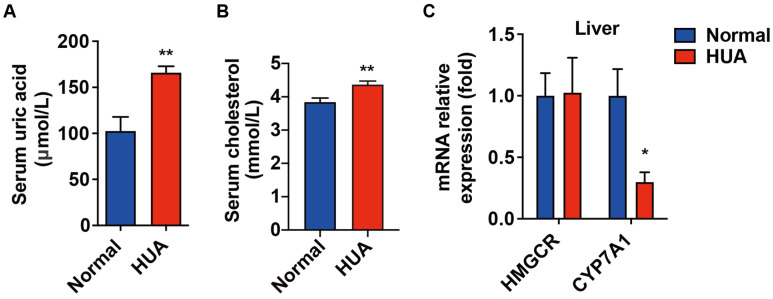
Elevated serum cholesterol level was related to down-regulated CYP7A1 in hyperuricemia mice. Normal control C57BL/6J mice group (Normal), PO-and adenine-induced hyperuricemia mice group (HUA). (**A**) Serum uric acid level. (**B**) Serum cholesterol level. (**C**) mRNA levels of hepatic HMGCR and CYP7A1. Data are presented as mean ± S.E.M. * *p* < 0.05, ** *p* < 0.01 vs. hyperuricemia mice group.

As shown in Figure 1A,B, compared with the normal group, PO and adenine treatment significantly increased the serum uric acid and cholesterol levels by 61.8% and 15.7%, respectively, in the hyperuricemia group. The high uric acid level did not affect the expression of cholesterol synthesis enzyme 3-hydroxy-3-methylglutaryl-CoA reductase (HMGCR), but reduced expression of CYP7A1 by 70.2% in the liver of hyperuricemia mice (Figure 1C). These data suggest that the dysfunction of cholesterol metabolism induced by hyperuricemia may be related to the conversion process of cholesterols to bile acid.

### 3.2. Uric Acid-Accelerated Atherosclerosis Progress in ApoE^−/−^ Mice with Elevated Serum Cholesterol Level, Decreased Hepatic Bile Acid Level. The Effects at Least in Part from Activation of FXR and Down-Regulation of CYP7A1 in the Liver

#### 3.2.1. Aortic Lesions Increased in PO-induced Hyperuricemic ApoE^−/−^ Mice

**Figure 2:**
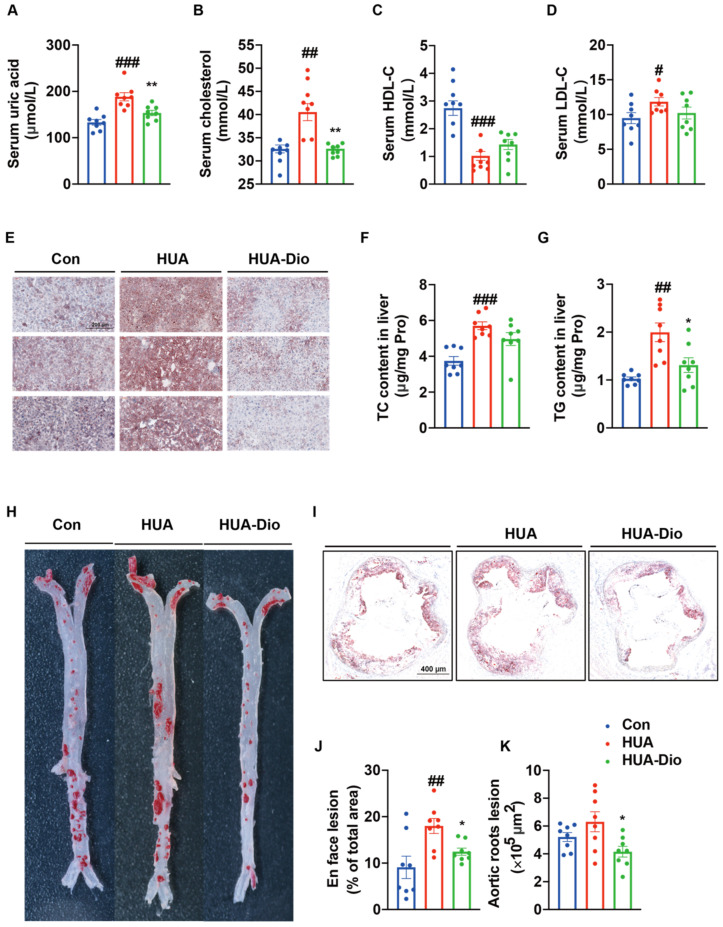
Dioscin alleviated hyperuricemia-aggravated atherosclerosis and dysfunction of cholesterol metabolism in ApoE^−/−^ mice. Control ApoE^−/−^ mice group (Con), hyperuricemic ApoE^−/−^ mice group (HUA), dioscin treatment hyperuricemic ApoE^−/−^ mice group (HUA-Dio). (**A**) Serum uric acid level. (**B**) Serum cholesterol level. (**C**) Serum HDL-C level. (**D**) Serum LDL-C level. (**E**) Oil Red O staining in the livers of mice. (**F**) TC contents in liver. (**G**) TG contents in liver. (**H**) Oil Red O staining of en face aortal lesion. (**I**) Oil Red O staining of lesion area in aortic root. (**J**) Quantification of en face aortal lesion. (**K**) Quantification of lesion. Data are presented as mean ± S.E.M. ^#^ *p* < 0.05, ^##^ *p* < 0.01, ^###^ *p* < 0.001 vs. control ApoE^−/−^ mice group; * *p* < 0.05, ** *p* < 0.01 vs. hyperuricemic ApoE^−/−^ mice group.

The APOE^−/−^ mice spontaneously exhibited atherosclerosis after the 3-month HCD diet. A PO-treated HCD-fed ApoE^−/−^ mice were used to test the acceleration effect of hyperuricemia on early atherosclerotic plaque progression. Aortic lesions were determined in HCD-fed ApoE^−/−^ mice with or without PO (250 mg/kg/d, intraperitoneally injection). After 3-month treatment, compared with the control ApoE^−/−^ mice group, the en face aortic lesions in hyperuricemic ApoE^−/−^ mice increased by 98.1% (Figure 2H,J). Consistent with the change in en face aortic lesions, the lesions area and lipid deposition in aortic roots (Figure 2I,K) significantly increased in the hyperuricemic ApoE^−/−^ mice group (area: (6.3 ± 0.7) × 10^5^ μm^2^) compared with the control ApoE^−/−^ mice group (area: (5.3 ± 0.9) × 10^5^ μm^2^). These data suggest that uric acid accelerated the atherosclerosis process in ApoE^−/−^ mice.

#### 3.2.2. Elevation of Serum Cholesterol Level was Aggravated in ApoE^−/−^ Mice under Hyperuricemic Condition

Compared with the control ApoE^−/−^ mice group, the serum levels of uric acid and TC were significantly increased by 41.5% and 18.6%, respectively, in the PO-treated hyperuricemic ApoE^−/−^ mice group (Figure 2A,B). In addition, compared with the control ApoE^−/−^ mice group, PO administration also significantly increased the serum LDL-C level (70.9%) and decreased the HDL-C level (24.9%) (Figure 2C,D). We also determined the hepatic TC and TG levels, and the results showed that PO treatment significantly increased hepatic TC and TG levels by 52.5% and 94.6%, respectively (Figure 2F,G). Oil Red O staining revealed that the marked hepatic steatosis was aggravated in the hyperuricemic ApoE^−/−^ mice group (Figure 2E).

#### 3.2.3. Hepatic Bile Acid Levels Decreased in PO-induced Hyperuricemic ApoE^−/−^ Mice, Associated with Decrease in Ileal-Conjugated Bile Acid Levels

Bile acid synthesis and excretion is a critical pathway in cholesterol homeostasis. We further determine the bile acid changes in hyperuricemic ApoE^−/−^ mice by an LC-MS-based targeted metabolomics approach.

**Figure 3:**
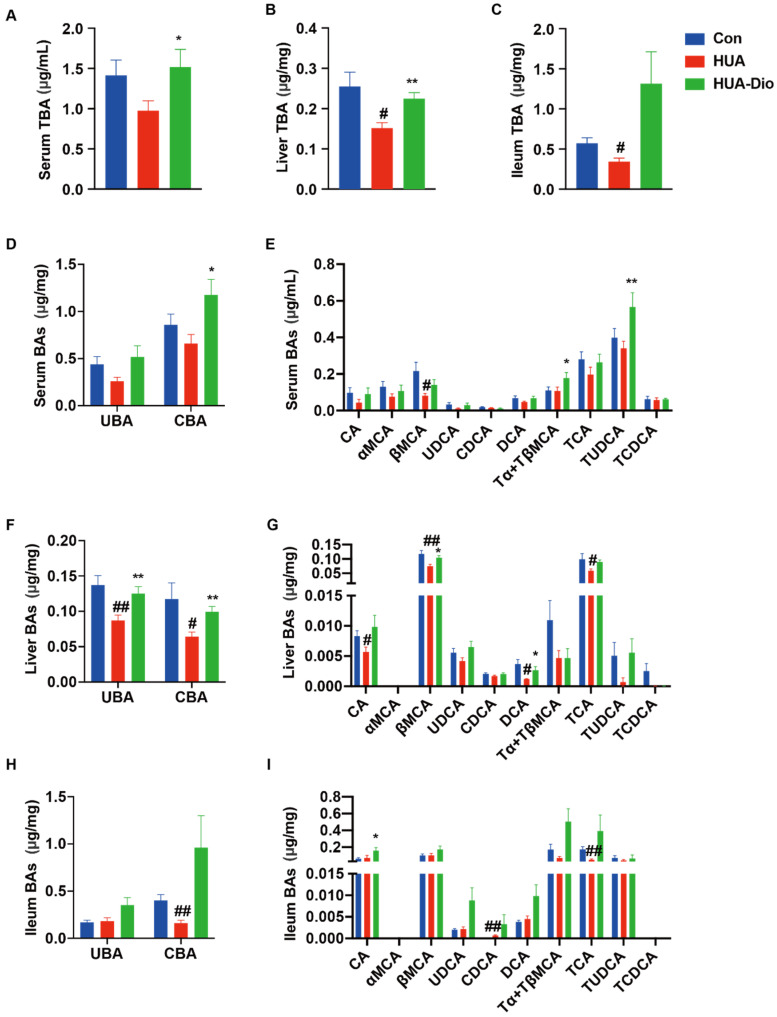
The effect of dioscin on the dysfunction of bile acid metabolism in hyperuricemic ApoE^−/−^ mice. Control ApoE^−/−^ mice group (Con), hyperuricemic ApoE^−/−^ mice group (HUA), dioscin treatment hyperuricemic ApoE^−/−^ mice group (HUA-Dio). (**A**–**C**) The levels of TBA in serum, liver, and ileum. (**D**) The levels of UBA and CBA in serum. (**E**) The bile acid profile in serum. (**F**) The levels of UBA and CBA in liver. (**G**) The bile acid profile in liver. (**H**) The levels of UBA and CBA in ileum. (**I**) The bile acid profile in ileum. Data are presented as mean ± S.E.M. ^#^ *p* < 0.05, ^##^ *p* < 0.01 vs. control ApoE^−/−^ mice group; * *p* < 0.05, ** *p* < 0.01 vs. hyperuricemic ApoE^−/−^ mice group.

Compared with the control ApoE^−/−^ mice, there was no significant change in the contents of serum total bile acid (TBA), unconjugated bile acid (UBA), and conjugated bile acid (CBA) in the hyperuricemic ApoE^−/−^ mice (Figure 3A,D). The contents of hepatic TBA, UBA, and CBA decreased by 43.7%, 36.6%, and 45.2%, respectively (Figure 3B,F), and the contents of ileal TBA and CBA decreased by 39.9% and 59.9%, respectively, in the hyperuricemic ApoE^−/−^ mice (Figure 3C,H).

We further detected the bile acid profile in different tissues. The contents of conjugated bile acids (TUDCA, TCA, TCDCA, TαMCA, and TβMCA), unconjugated bile acids (CA, αMCA, βMCA, UDCA, and CDCA), and secondary bile acids (DCA and LCA) were measured. Compared with the control ApoE^−/−^ mice, the content of serum βMCA markedly decreased by 62.3% (Figure 3E); the contents of hepatic CA, βMCA, DCA, and TCA significantly decreased by 31.7%, 36.8%, 67.6%, and 40.5%, respectively (Figure 3G); and the content of ileal TCA markedly decreased by 72.1% in PO-treated hyperuricemic ApoE^−/−^ mice (Figure 3I).

These results indicated that the conversion efficiency from cholesterol to bile acid decreased under hyperuricemic conditions, leading to dysfunction of cholesterol metabolism.

#### 3.2.4. Hepatic FXR Was Activated and CYP7A1 Was Down-Regulated in PO-Induced Hyperuricemic ApoE^−/−^ Mice

CYP7A1 is the rate-limiting enzyme of bile acid synthesis in the liver. The expression and activity of CYP7A1 determine the conversion rate of cholesterols to bile acid. The expression of CYP7A1 is mainly regulated through two pathways in the gut–liver axis: FXR-small heterodimer partner (SHP) [21] and fibroblast growth factor (FGF15) -fibroblast growth factor receptor 4 (FGFR4) [22]. In liver, FXR can directly activate the transcription of SHP, and the latter negatively regulates CYP7A1 and other CYPs (oxysterol 7α-hydroxylase (CYP7B1), sterol 12α-hydroxylase (CYP8B1), etc.) expression. In ileum, FXR activation promotes the release of FGF15, which enters the liver via enterohepatic circulation, binds to FGFR4, and then represses hepatic CYP7A1.

**Figure 4:**
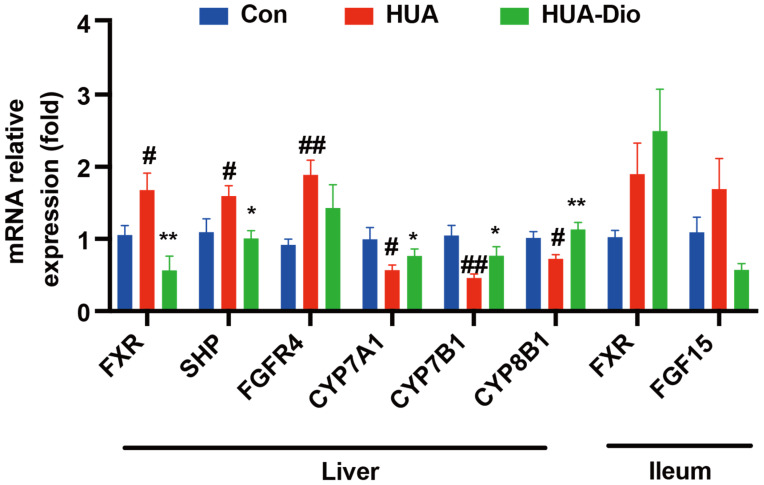
Expression of genes involved in bile acids synthesis in liver and ileum of ApoE^−/−^ mice. Control ApoE^−/−^ mice group (Con), hyperuricemic ApoE^−/−^ mice group (HUA), dioscin-treated hyperuricemic ApoE^−/−^ mice group (HUA-Dio). Data are presented as mean ± S.E.M. ^#^ *p* < 0.05, ^##^ *p* < 0.01 vs. control ApoE^−/−^ mice group, * *p* < 0.05, ** *p* < 0.01 vs. hyperuricemic ApoE^−/−^ mice group.

As shown in Figure 4, compared with the control ApoE^−/−^ mice, the mRNA levels of hepatic FXR, SHP, and FGFR4 were significantly upregulated by 59.0%, 45.7%, and 105.4%, respectively, in hyperuricemic ApoE^−/−^ mice. The mRNA levels of hepatic CYP7A1, CYP7B1, and CYP8B1 were significantly downregulated by 42.8%, 52.6%, and 28.6%, respectively. The mRNA levels of ileal FXR and FGF15 showed tendencies to increase in hyperuricemic ApoE^−/−^ mice (Figure 4).

These results indicated that the decrease in conversion efficiency under hyperuricemic conditions is at least in part related to the FXR-CYP7A1 axis, thereby disturbing the balance between cholesterols and bile acids.

### 3.3. Dioscin Alleviated Hyperuricemia-Aggravated Atherosclerosis and Improved the Conversion of Cholesterols to Bile Acid in the Liver of ApoE^−/−^ Mice

#### 3.3.1. Dioscin Alleviated Hyperuricemia-Aggravated Atherosclerosis and Dysfunction of Lipid Metabolism

After treatment with dioscin (100 mg/kg/d) in hyperuricemic ApoE^−/−^ mice for 3 months, Oil Red O staining demonstrated that dioscin reduced atherosclerotic lesions in both aortas (30.8%) and aortic roots (34.1%) compared with untreated hyperuricemic ApoE^−/−^ mice (Figure 2H–K). These results suggested that dioscin prevented the atherosclerosis progress that was exacerbated by hyperuricemia.

Dioscin treatment markedly reduced serum uric acid and cholesterol levels by 18.6% and 23.1%, respectively, compared with the control hyperuricemic ApoE^−/−^ mice group (Figure 2A,B). However, there were no significant changes in the serum LDL-C and HDL-C levels (Figure 2C,D). In the liver, dioscin treatment significantly decreased the TG level by 34.3% but had no significant effect on the TC level (Figure 2G). Oil Red O staining revealed marked hepatic steatosis in the liver of the hyperuricemic ApoE^−/−^ mice group, which was attenuated in the dioscin-treated group (Figure 2E). These results indicated that dioscin improved uric acid and dysfunction of lipid metabolism.

#### 3.3.2. Dioscin Administration Modulated the Dysfunction of Bile Acid Profile in Hyperuricemic ApoE^−/−^ Mice

Dioscin administration dramatically elevated the serum content of TBA and CBA by 55.4% and 78.2%, respectively (Figure 3A,D), and increased the hepatic contents of TBA, UBA, and CBA by 48.3%, 30.4%, and 54.5%, respectively (Figure 3B,F) compared with the control hyperuricemic ApoE^−/−^ mice. However, no significant difference was observed in ileum (Figure 3C,H). We further analyzed the profile of bile acid in different tissues. Dioscin treatment significantly increased the serum contents of TUDCA, TαMCA, and TβMCA (Figure 3E), associated with increased levels of hepatic βMCA and DCA (Figure 3G). However, dioscin treatment had no effect on the bile acid profile in the ileum (Figure 3I). These results demonstrated that dioscin administration modulated the hepatic bile acid profile in hyperuricemic ApoE^−/−^ mice.

#### 3.3.3. Dioscin Treatment Inhibited Hepatic FXR Signaling

Compared with the control hyperuricemic ApoE^−/−^ mice, treatment with dioscin significantly decreased the mRNA levels of hepatic FXR and SHP by 66.3% and 36.8%, respectively, while there was no significant difference in the level of FGFR4. Moreover, the mRNA levels of hepatic CYP7A1, CYP7B1, and CYP8B1 significantly increased by 34.5%, 67.1%, and 56.2%, respectively. However, there were no significant differences in the mRNA levels of intestinal FXR and FGF15 between the dioscin-treated and the control groups (Figure 4). These results indicated that dioscin treatment promoted the conversion efficiency from cholesterols to bile acid by inhibiting FXR, thereby maintaining cholesterol homeostasis.

### 3.4. Dioscin Inhibited FXR through Its Metabolite Tigogenin

**Figure 5:**
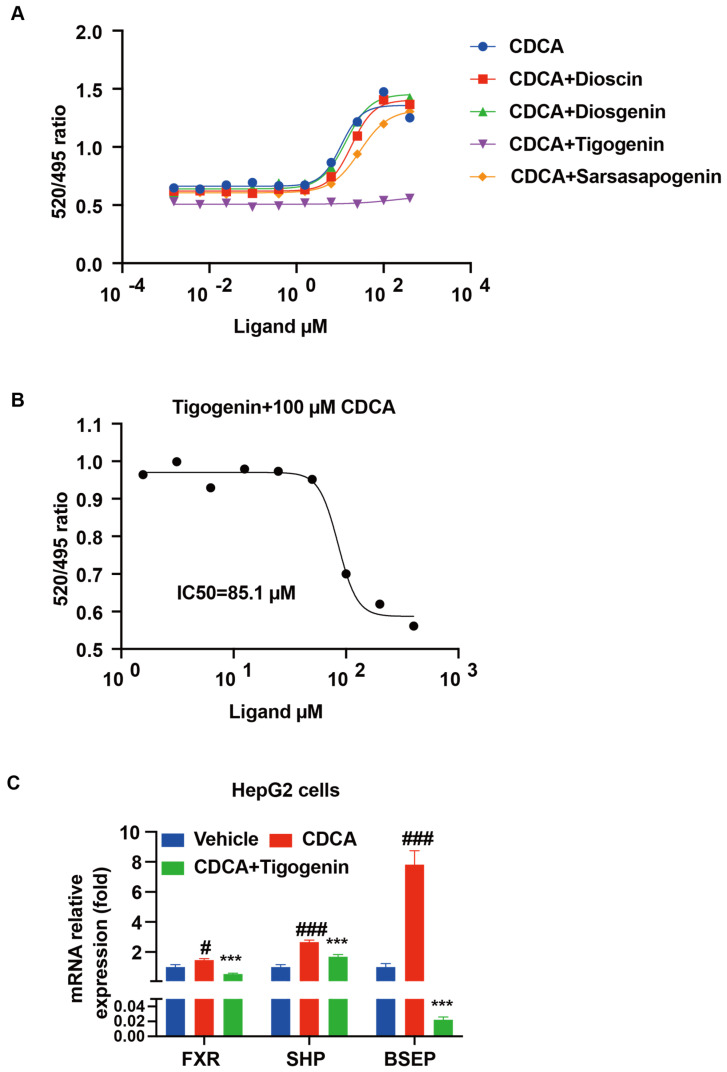
Effect of dioscin and its metabolites on FXR activation by CDCA: (**A**) Activation of FXR by CDCA in the presence of 100 μM ligands (dioscin and its metabolites) to demonstrate the reversibility of binding. (**B**) TR-FRET FXR coactivator recruitment assay to assess whether tigogenin is a functional antagonist to CDCA (100 μM) -mediated activation of FXR. (**C**) FXR target genes mRNA expression in HepG2 cells after treatment with CDCA alone or 20 μM tigogenin with CDCA for 24 h. Data are presented as mean ± S.E.M. ^#^ *p* < 0.05, ^###^ *p* < 0.001 vs. vehicle group, *** *p* < 0.001 vs. CDCA group. GraphPad Prism version 8.0 (GraphPad Software, San Diego, CA, USA) was used to fit the experimental data points to curves and to calculate EC_50_ and IC_50_ values.

The above results demonstrated that the hepatic FXR signaling was suppressed by dioscin. We hypothesized that dioscin or its metabolites may act as FXR antagonists. We performed the TR-FRET FXR coactivator assay to test our hypothesis. Using this system, the EC_50_ of CDCA, a natural ligand of FXR, was 10.5 μM. Dioscin and its metabolites (diosgenin and sarsasapogenin) did not show agonistic actions on the activation of FXR by CDCA (Figure 5A). However, tigogenin, a metabolite of dioscin and an epimer of sarsapogenin, showed a strong competitive inhibition effect on the CDCA (100 μM) -mediated recruitment of SRC2 to FXR, with an IC_50_ of 85.1 μM (Figure 5B).

In addition, the expression levels of FXR target genes in HepG2 cells were determined with or without tigogenin treatment. Consistently, the mRNA levels of FXR and its target genes SHP and bile salt export pump (BSEP) were approximately 0.5-fold, 1.6-fold, and 6.8-fold higher, respectively, in the CDCA-treated group than in the control group. Compared to treatment with 50 μM CDCA alone, the mRNA levels of FXR, SHP, and BSEP significantly decreased by 0.6-fold, 0.4-fold, and 1.0-fold, respectively, in the presence of 20 μM tigogenin (Figure 5C). These results indicated that tigogenin may be the active metabolite of dioscin on suppression of FXR, modulating the conversion of cholesterols to bile acid.

### 3.5. Treatment with Dioscin-Enriched Preparation Ameliorates Hypercholesterolemia in Hyperuricemia Patients

We used a dioscin-enriched preparation, whose commercial name is Di’ao Xinxuekang, a post-marketing herbal capsule in China and the Netherlands, to explore the activity of dioscin on hyperuricemia-induced dysfunction of cholesterol metabolism. The active ingredient (Dioscin) of DA is extracted from the rhizomes of *Dioscorea panthaica* Prain and Burkill. As literature reported [23], after hydration, ring cyclization, and deglycosylation by intestinal microflora and hepatic drug enzyme, spirostanols in DA were metabolized to dioscin or its aglycon as the main metabolites commonly found in serum.

#### 3.5.1. Description of Patients and Interventions

**Figure 6:**
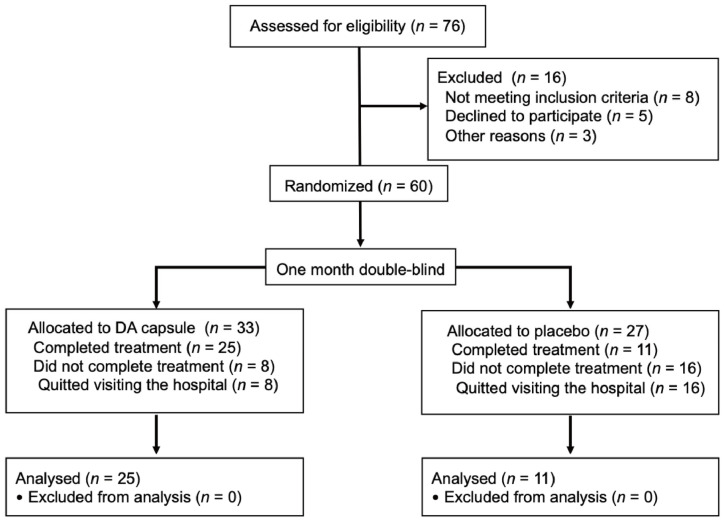
Flow Diagram of hyperuricemia subjects and interventions.

A total of 60 participants aged 18 to 60 years old whose serum uric acid levels were higher than 420 μmol/L were enrolled. The participants were randomized into two arms, which were the control group (*n* = 27) and the DA group (*n* = 33). The DA group received 600 mg of DA per day for one month. A total of 8 out of 33 patients in the DA group and 16 out of 27 patients in the control group did not complete the study. Twenty-five patients (75.8%) in the DA group and eleven patients (40.7%) in the control group were included in the analysis of the primary endpoint (Figure 6).

#### 3.5.2. Demographic and Baseline Characteristics

The baseline demographics and clinical characteristics of the participants are shown in Table 1. There was no significant difference between the DA group and the control group at baseline. Mean baseline serum uric acid was 497.45 ± 43.73 μmol/L in the control group and 514.22 ± 57.33 μmol/L in the DA group, respectively. The mean baseline cholesterol level was 4.89 ± 0.87 mmol/L in the control group and 5.08 ± 1.01 mmol/L in the DA group, respectively.

#### 3.5.3. Serum Uric Acid and Cholesterol Analysis

**Figure 7:**
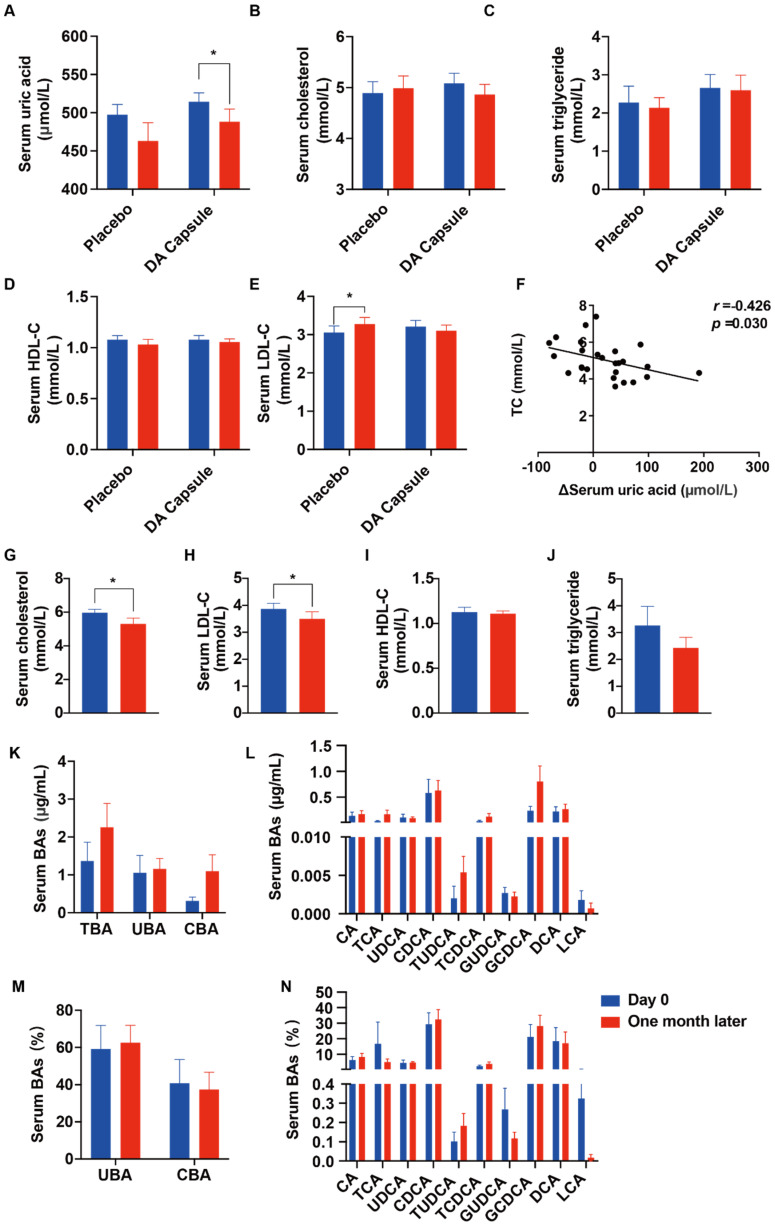
Effect of DA capsule on serum lipid levels and bile acid profile in hyperuricemia subjects: (**A**–**E**) Effects of DA capsule on serum uric acid and serum lipid levels in hyperuricemia subjects. (**F**) Correlation between changes in serum uric acid levels and total cholesterol levels. ΔSUA = Day 0–one month later. (**G**–**J**) Effects of DA capsule on serum lipid levels in hyperuricemia subjects (TC > 5.17 mmol/L at baseline, *n* = 11). Δ = Day 0–one month later. (**K**,**L**) The contents of serum bile acids in hyperuricemia subjects (TC > 5.17 mmol/L at baseline, *n* = 11). (**M**,**N**) The proportions of serum bile acids in hyperuricemia subjects (TC > 5.17 mmol/L at baseline, *n* = 11). Data are presented as mean ± S.E.M. * *p* < 0.05 vs. Day 0.

DA administration led to decreased levels of serum uric acid from 514.22 ± 57.33 to 488.24 ± 82.61 μmol/L (*p* = 0.034) during the treatment period. (Figure 7A). However, there was no significant change in the serum uric acid level f in the control group (*p* = 0.101). There was no significant difference in the serum lipid levels between the DA group and the control (Figure 7B–E). LDL-C levels had a significant increase from the baseline in the control group, but not in the DA group (Figure 7C). Furthermore, a subgroup analysis was carried out to evaluate the relationship between UA and cholesterol. The result indicated that patients with a TC > 5.17 mmol/L at baseline showed significant reductions (*p* = 0.010) in TC and LDL-C levels (*p* = 0.020) from baseline in the DA group (Figure 7G,H). However, there was no significant reduction in the serum HDL-C and TG levels (Figure 7I,J). Of note, Pearson’s analysis showed that the change in serum uric acid levels was negatively correlated with cholesterol levels (*r* = −0.462, *p* = 0.030) after DA treatment for one month (Figure 7F).

### 3.6. The BA Profile Changes after Oral Administration of Dioscin-Enriched Preparation

To determine the effect of DA treatment on the bile acid profile, an LC-MS approach was used to analyze the bile acid in the serum of hyperuricemia patients. As shown in Figure 7K–N, in the DA-treated subjects with a serum uric acid TC > 5.17 mmol/L at baseline, there was a tendency for the elevation of serum TBA and CBA (TUDCA and GCDCA, etc.) after the treatment.

The clinical data confirmed the relationship between uric acid and cholesterol, and further clarified that tigogenin was the active metabolite of dioscin on suppression of FXR, modulating the conversion of cholesterols to bile acid.

## 4. Discussion

Although hyperuricemia is not a disease, it is known that long-term elevated uric acid levels are associated with gout, hypertension, diabetes, renal diseases, and cardio-cerebral vascular diseases. Clinical studies have shown that every 1 mg/dl increase in serum uric acid level was associated with a 23% higher incidence of atherosclerosis [24]. As a result, several clinical guidelines have listed hyperuricemia as one of the important risk factors for cardiovascular diseases in recent years [5]. However, it has been difficult to implement effective clinical interventions for hyperuricemia-related atherosclerosis because the underlying mechanisms remain unclear.

It is estimated that the prevalence of lipid abnormalities in the overall hyperuricemia individual was approximately 51% [25]. The clinical trials reported that XOI treatment did not play a beneficial role in preventing atherosclerosis [7], which indicates that uric acid is not a direct cause of cardiovascular disease. Hyperuricemia is associated with abnormal lipid metabolism, especially with hypertriglyceridemia and hypercholesterolemia [26]. Given that hypercholesterolemia is a well-documented risk factor for cardiovascular diseases [27], we deemed it is necessary to further investigate the mechanism of hyperuricemia-related dysfunction of cholesterol metabolism, aiming at providing guidance for precise therapy.

We found in this study that hyperuricemia induced the activation of FXR, which lowered the conversion of cholesterols converted to bile acids, causing increased cholesterol levels and subsequent atherosclerosis. Meanwhile, we found that Tigogenin, a metabolite of dioscin, inhibited the activation and upregulation of FXR by hyperuricemia, and improved cholesterol homeostasis. This had been supported by a small-scale clinical trial. This study shed light on the precise treatment of the hyperuricemia-related abnormality of cholesterol metabolism.

Dysfunction of cholesterol metabolism is linked to other primary diseases with various biochemical and pathological mechanisms. Hypothyroidism modifies the content and signaling of lipids by a reduction in the expression of perilipin A and proliferator-activated receptor δ [28]. Patients with chronic kidney disease have downregulation of the expression of hepatic apolipoprotein A-I and lecithin cholesterol acyltransferase, which results in impaired high-density lipoprotein-mediated cholesterol uptake from vascular tissue [29]. Patients with nonalcoholic fatty liver disease (NAFLD) or nonalcoholic steatohepatitis showed significant up-regulation of protease-activated receptor 2, accompanied by increasing de novo lipogenesis, and leading to the dysfunction of cholesterol metabolism [30].

Dysfunction of cholesterol metabolism occurs with different mechanisms [13]. Some clinical studies have shown that the effect of statins on reducing the risk of cardiovascular diseases varied tremendously in treating different types of hypercholesterolemia due to different pathogeneses; although, statins do lower serum cholesterol levels consistently [31].

For example, in a NAFLD patient, statins significantly lowered the cardiovascular risks without unexpected adverse events [32]. By contrast, a clinical trial showed that statins had no significant effect on the primary composite end point of death from cardiovascular causes, nonfatal myocardial infarction, or nonfatal stroke in hemodialysis patients [33]. Therefore, the risk of cardiovascular disease can only be effectively reduced by pertinent mechanism-based treatments for hypercholesterolemia. 

We demonstrated in this study for the first time that the synthesis and absorption of cholesterols did not change significantly in the hyperuricemic state. However, the hepatic FXR was abnormally activated, which caused the inhibition of CYP7A1 expression and lowered the conversion rate of cholesterols to bile acids. This led to increased cholesterol levels and subsequent atherosclerosis. The abnormal cholesterol metabolism in the hyperuricemic state was mainly related to the dysregulation of the FXR-CYP7A1 axis. Current commonly used medications for hyperlipidemia include, but are not limited to: statins that lower cholesterol synthesis; ezetimibe, which inhibits cholesterol absorption; and proprotein convertase subtilisin/kexin type 9, which lowers LDL receptors. The pharmacological actions of these medications are not through the FXR-CYP7A1 axis. Therefore, it is impossible to provide precise therapy for hyperuricemia-related dysfunction of cholesterol metabolism by using these drugs.

Dioscin is widely distributed in herbal medicine and foodstuff, with anti-fungal, anti-viral [34], hepatoprotective [35], and anti-hyperuricemia [16] pharmacological activities. In this study, we found that oral dioscin lowered serum uric acid and cholesterol levels, and delayed the progression of atherosclerotic plaque formation.

As a spirostane glycoside, after gut microbiota and liver metabolism, dioscin would be converted to tigogenin, sarsasapogenin, and diosgenin, which contributed to its bioactivity in vivo [23]. Within the metabolites of dioscin, tigogenin showed inhibition of the effect on FXR activation with an IC_50_ of 85.1 μM. In vitro activity screening assay followed by in vivo activity study showed that tigogenin significantly suppressed FXR activation and increased CYP7A1 levels, and enhanced the conversion of cholesterols to bile acids. Interestingly, tigogenin and sarsasapogenin, two main metabolites of dioscin, are epimers that structurally differ only in the spatial arrangement of the 25-methyl group. However, their actions of activating FXR are tremendously different.

Further clinic study revealed dioscin significantly improved cholesterol metabolism in subjects with hyperuricemia after one-month oral administration. Our previous research reported that dioscin could decrease serum uric acid levels through increasing renal and intestinal uric acid excretion [16]. The current study further clarified the therapeutic action of dioscin on hyperuricemia-related dysfunction of cholesterol metabolism. Dioscin is abundant in many natural products. It is apparently a potential drug and/or medicinal food for hyperuricemia-related dysfunction of cholesterol metabolism.

In this paper, the initial design of the clinical research was based on the evaluation of the uric acid-lowering activity of dioscin, and hyperuricemia was defined as the key inclusion criteria of the target population, which led to the small sample size in the subgroup analyses. Our next step is to increase the recruitment of individuals with hyperuricemia as well as hypercholesterolemia to further the prevention effect and mechanisms of dioscin on hyperuricemia-induced dysfunction of cholesterol metabolism. 

## Figures and Tables

**Table 1:** Study subjects’ demographic data.

Parameter (S.D.)	Baseline			One Month Later		
	Control (*n* = 11)	DA Capsule (*n* = 25)	*p*	Control (*n* = 11)	DA Capsule (*n* = 25)	*p*
Age (years)	40.31 ± 9.54	40.15 ± 8.88	0.961	40.44 ± 2.13	40.15 ± 8.882	0.961
ALT (U/L)	31.16 ± 19.22	32.87 ± 17.29	0.770	29.30 ± 20.76	32.71 ± 17.85	0.579
AST (U/L)	31.87 ± 28.52	20.87 ± 5.35	0.160	21.77 ± 6.26	22.72 ± 7.94	0.693
Blood urea nitrogen (mmol/L)	5.72 ± 1.08	5.84 ± 1.09	0.752	5.53 ± 0.88	5.23 ± 0.92 *	0.319
BMI (kg/m^2^)	28.83 ± 3.42	28.90 ± 5.19	0.970	28.78 ± 3.66	28.29 ± 5.75	0.817
Diastolic BP (mm Hg)	88.10 ± 9.96	83.19 ± 16.25	0.400	85.30 ± 8.49	81.13 ± 16.05	0.457
Fasting glucose (mmol/L)	5.24 ± 0.69	5.27 ± 0.93	0.925	5.19 ± 0.81	5.35 ± 0.69	0.560
HDL-C (mmol/L)	1.08 ± 0.16	1.08 ± 0.21	0.996	1.03 ± 0.20	1.06 ± 0.15	0.650
Heart rate (/min)	67.17 ± 8.08	69.20 ± 11.79	0.603	68.08 ± 8.45	71.00 ± 10.46	0.420
Height (cm)	177.10 ± 5.71	176.59 ± 7.56	0.800	177.10 ± 5.71	176.59 ± 7.56	0.800
Hemoglobin (g/L)	150.40 ± 9.83	158.16 ± 9.24	0.016	151.53 ± 6.74	157.40 ± 7.77	0.020
LDL-C (mmol/L)	3.05 ± 0.66	3.21 ± 0.82	0.539	3.27 ± 0.69 *	3.10 ± 0.76	0.470
Serum creatinine (μmol/L)	83.91 ± 14.03	87.87 ± 11.38	0.326	85.67 ± 14.51	85.66 ± 10.41	0.996
Systolic BP (mm Hg)	132.90 ± 16.45	127.56 ± 17.55	0.448	123.00 ± 18.74 *	125.25 ± 22.05	0.971
Total cholesterol (mmol/L)	4.89 ± 0.87	5.08 ± 1.01	0.541	4.99 ± 0.93	4.86 ± 1.02	0.700
Triglycerides (mmol/L)	2.27 ± 1.65	2.66 ± 1.79	0.502	2.14 ± 1.03	2.60 ± 2.02	0.417
Uric acid (μmol/L)	497.45 ± 43.73	514.22 ± 57.33	0.394	463.15 ± 78.69	488.24 ± 82.61 *	0.401
Urine creatinine (mg/dL/24 h)	15.44 ± 2.12	17.34 ± 7.19	0.331	15.10 ± 3.54	20.05 ± 12.29	0.109
Urine uric acid (mg/24 h)	631.68 ± 163.55	773.28 ± 344.18	0.153	639.81 ± 212.57	851.93 ± 421.22	0.067
Urine volume (L)	1.98 ± 0.70	2.12 ± 0.79	0.603	2.00 ± 0.57	2.20 ± 1.02	0.482
VLDL (mmol/L)	0.76 ± 0.39	0.80 ± 0.35	0.745	0.68 ± 0.24	0.71 ± 0.46	0.857
WBC count (×10^9^/mL)	6.90 ± 1.31	6.22 ± 1.47	0.148	7.37 ± 1.36	6.36 ± 1.28	0.024
Weight (kg)	90.10 ± 8.27	91.50 ± 21.75	0.829	89.60 ± 8.54	90.07 ± 22.74	0.944
γGGT (U/L)	38.17 ± 27.44	47.10 ± 31.19	0.369	37.85 ± 23.99	45.90 ± 36.31	0.452

BMI, body mass index; BP, blood pressure; HDL-C, high-density lipoprotein cholesterol; LDL-C, low-density lipoprotein cholesterol; VLDL, very low-density lipoprotein; WBC, leukocyte; AST, aspartate transaminase; ALT, cereal third transaminase; γGGt, γ-glutamyl transpeptidase. Data are presented as mean ±S.D. *p* values are analyzed for differences between the control and DA groups by the unpaired *t*-test. * *p*  <  0.05 vs. parameter level at baseline in each respective group.

## Data Availability

Not applicable.

## Supplementary Materials

The following supporting information can be downloaded at: https://www.mdpi.com/article/10.3390/nu14091983/s1, Supplementary Table S1. The gradient elution of mobile phase for bile acid profile analysis; Supplementary Table S2. Primer sequences for Real-time PCR studies on mice; Supplementary Table S3. Primer sequences used for Real-time PCR studies on HepG2 cells.
