# Supplementary material for: Dioscin Ameliorates Hyperuricemia-Induced Atherosclerosis by Modulating of Cholesterol Metabolism through FXR-Signaling Pathway
Source: Nutrients. 2022 May 9;14(9):1983. doi: 10.3390/nu14091983 (PMC9101489; doi:10.3390/nu14091983)
Supplement: Supplementary file 1 [file nutrients-14-01983-s001.zip › nutrients-1701840-supplementary.pdf]

Supplementary

Supplementary Table Legends:

Supplementary Table S1. The gradient elution of mobile phase for bile acid profile analysis

Supplementary Table S2. Primer sequences for Real-time PCR studies on mice

Supplementary Table S3. Primer sequences used for Real-time PCR studies on HepG2 cells

Supplementary Table S1. The gradient elution of mobile phase for bile acid profile analysis

Time (min)	Flow rate (mL/min)	A (%)	B (%)
0	0.4	95	5
5	0.4	95	5
5.5	0.4	80	20
14	0.4	80	20
19	0.4	70	30
24	0.4	70	30
24.5	0.4	60	40
27.5	0.4	60	40
30.5	0.4	20	80
32.5	0.4	20	80
33.5	0.4	95	5
35	0.4	95	5

Supplementary Table S2. Primer sequences for Real-time PCR studies on mice

Gene name	Forward sequence (5' to3')	Reverse sequence (5' to3')
FXR	CCCCTGCTTGATGTGCTAC	CGTGGTGATGGTTGAATGTC
SHP	CGATCCTCTTCAACCCAGATG	AGGGCTCCAAGACTTCACACA
FGF15	TGTTTCACCGCTCCTTCTTT	TCTACATCCTCCACCATCCTG
FGFR4	GCATCTTTCAGGGGACACCA	TTGTACCAGTGACGACCACG
CYP7A1	CTGGGCTGTGCTCTGAAGT	GGGAGTTTGTGATGAAGTGGA
CYP8B1	ACAGCGTGATGGAGGAGAGT	AGGGGAAGAGAGCCACCTTA
CYP7B1	TGAGGTTCTGAGGCTGTGC	TGGAGGAAAGAGGGCTACAA
HMGCR	AGCTTGCCCGAATTGTATGTG	TCTGTTGTGAACCATGTGACTTC
GAPDH	TGTGTCCGTCGTGGATCTGA	CCTGCTTCACCACCTTCTTGAT

Supplementary Table S3. Primer sequences used for Real-time PCR studies on HepG2 cells

Gene name	Forward sequence (5' to3')	Reverse sequence (5' to3')
FXR	TACATGCGAAGAAAGTGTCAAGA	ACTGTCTTCATTCACGGTCTGAT
SHP	TCAAGTCCATTCCGACCAGC	AAGAAGGCCAGCGATGTCAA
BSEP	TGATCCTGATCAAGGGAAGG	GGCAAACAACACTGGTTCCT
GAPDH	CCAGCAAGAGCACAAGAGGA	GAGATTCAGTGTGGTGGGGG
